# How ex-employee citizenship behavior is generated: From the perspective of legacy identification

**DOI:** 10.3389/fpsyg.2022.947142

**Published:** 2022-12-02

**Authors:** Zehui Tian, Qinghong Yuan, Shanshan Qian, Yanhong Guo

**Affiliations:** ^1^Business School, Nankai University, Tianjin, China; ^2^Business School, Guangdong University of Foreign Studies, Guangzhou, China; ^3^Business School, Jilin Normal University, Siping, China

**Keywords:** legacy identification, perceived organizational prestige, perceived insider status, identity salience, ex-employee citizenship behavior

## Abstract

The termination of employment is not the end of an organization–employee relationship. As ex-employees can provide various benefits to their former organizations, and a large number of ex-employees have accumulated in enterprises because of increased employee mobility, research on ex-employees’ contribution behavior, and how it is generated are significant to organizations in making use of their ex-employees effectively and consequently improving organizational efficiency. Based on the research into organizational citizenship behavior, Study 1 extended the focus of organizational citizenship behavior research to include ex-employees, introducing the concept of ex-employee citizenship behavior. The measurement of ex-employee citizenship behavior was developed based on Hinkin’s tutorial. Using social identity theory, Study 2 discussed how ex-employee citizenship behavior is generated. A two-wave survey of 291 former employees was conducted. Hierarchical regression analysis and the bootstrap method were then applied to test the hypotheses. The results showed that legacy identification was positively related to ex-employee citizenship behavior. Furthermore, the interaction between perceived organizational prestige and perceived insider status was positively related to legacy identification. Perceived organizational prestige and perceived insider status were also indirectly and interactively related to ex-employee citizenship behavior through legacy identification. The positive relationship between legacy identification and ex-employee citizenship behavior was moderated by the cooperative relationship between the current and former organizations. Additionally, the indirect positive effect of the interaction between perceived organizational prestige and perceived insider status on ex-employee citizenship behavior through legacy identification is moderated by the cooperative relationship between the current and former organizations. The theoretical and practical implications of this study were discussed. Finally, the limitations of this study were presented alongside suggestions for future research.

## Introduction

Historically, employment termination has been considered the end of the relationship between organizations and ex-employees (Shipp et al., 2014). However, this view ignores the possibility that ex-employees may provide continuous value to their former organizations. Previous studies have confirmed that ex-employees benefit former organizations in many ways, such as by improving the latter’s creative performance (Godart et al., 2014), recommending business ideas (Carnahan and Somaya, 2013), transferring knowledge (Corredoira and Rosenkopf, 2010), improving organizational performance (Somaya et al., 2008), and enhancing firm influence (Dokko and Rosenkopf, 2010). McKinsey, for instance, was able to realize myriad business opportunities because of the McKinsey alumni network. Moreover, as employee mobility increases, companies accumulate an increasing number of former employees (Dachner and Makarius, 2022). According to the survey statistics of the Cyberspace Administration of China (2022), for example, the total turnover of employees in 12 Internet companies from July 2021 to March 2022 was 216,800. In addition, according to Ernst and Young Alumni (2022), the company has accumulated more than 1,000,000 ex-employees. These trends warrant further investigation of ex-employee behavior.

The majority of previous research on ex-employees’ contributions has been conducted in the field of organizational strategy, based on social network and social capital theory. Godart et al. (2014), for example, explained that the impact of key personnel loss can enhance former employers’ creative performance, as per the social network perspective. Carnahan and Somaya (2013) verified that ex-employees who find employment with former organizations’ buyers can bring these organizations additional business because of the relational advantages. Corredoira and Rosenkopf (2010) also posited that social ties caused by employee mobility may enhance knowledge transfer to the former organization. Dokko and Rosenkopf (2010) furthermore argued that mobility can affect firm influence through firm social capital. Somaya et al. (2008) also drew on social capital theory to argue that employee movement to cooperators could enhance a former firm’s performance. These studies provide sufficient evidence for the value of ex-employees; however, they place no conceptual value on personal choice and thus speak only to network positions and strengths. Lin (2001) posited that the value of a social network is potential. Ex-employees, even with the same social network position, will not make consistent choices to benefit their former organization. As such, personal choices play a key role in realizing these values. However, in contrast to the rich literature on organizational strategy, only two existing studies have elaborated on this topic from a personal perspective in the field of organizational behavior. In one of these studies, Herda and Lavelle (2011) found that organizational fairness predicts post-employment citizenship through perceived organizational support and commitment, based on social exchange theory (SET). Additionally, Iyer et al. (1997) found that both organizational and individual factors are related to ex-employees’ inclination to benefit their former firm, through organizational identification. Despite the merit of their findings, these studies have some limitations. First, both focused on ex-employees’ benefit inclination and failed to further examine their contribution behaviors, although it is human behaviors that directly lead to meaningful purposes. Second, both studies used business recommendations to indicate an inclination to benefit, ignoring other types of benefits such as knowledge transfers and talent recommendations. Third, these studies presented distinct causes, despite complex and interactive antecedents. And fourth, neither study explores the boundary conditions of generating ex-employee citizenship behavior.

To fill these research gaps, this study extends the concept of organizational citizenship behavior (OCB) to include ex-employees, to capture the various behaviors that could benefit their former organizations. In line with Organ (1988), this study defines ex-employee citizenship behavior (Ex-ECB) as self-discretionary behaviors, not directly or explicitly recognized by the formal reward system, which promotes the former organization’s effectiveness. This study also explores how Ex-ECB is generated based on social identity theory (SIT), which provides a valuable perspective for explaining individual behaviors (Ashforth and Mael, 1989). SIT suggests that the more a person identifies with an organization, the more he or she will actively contribute to it (Van Dyne et al., 1994). Previous studies maintain that some individuals still identify with their former organization, even after the cessation of employment with the organization; this is called legacy identification (LI) (Eury et al., 2018). This phenomenon can explain how Ex-ECB is generated. According to SIT, one of the most distinctive features of group life and intergroup relations is positive distinctiveness—a belief that “we” are better than “them” in every way. The sense of belonging in an organization is closely related to an individual’s self-concept through social classification (Pratt et al., 2021). As people often enhance their self-concepts (Hogg, 2006), perceived organizational prestige (POP) could influence LI by providing ex-employees with positive distinctiveness. In addition, Hogg (2006) holds that identification does not completely result from organizational prestige, which helps to define abstract human categories. Furthermore, the two parties’ relationship quality is the psychological basis of identification (Hogg, 2006). The interaction between POP and perceived insider status (PIS) is antecedental since PIS is a significant aspect in evaluating the relationship quality between ex-employees and their former organizations (Stamper and Masterson, 2002). In addition, the relationship between identification and subsequent behaviors is influenced by identity salience (Hogg, 2006; Marin et al., 2009). In the post-employment context, identity salience can be defined as a cooperative relationship (CR) between an employee’s current and former organizations (Campbell et al., 2012; Tian et al., 2022). When identity salience is present, LI is often regarded less threateningly and tends to be unsuppressed by the present organization (Bardon et al., 2015). Thus, the CR between the current and the former organization may moderate the relationship between LI and the resulting Ex-ECB.

**FIGURE 1:**
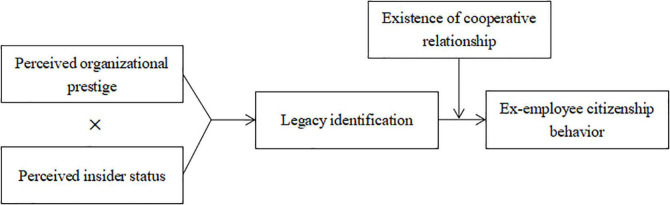
Hypothesized research model.

This study contributes to the existing literature in several ways. First, it extends OCB to include ex-employees and proposes the concept of Ex-ECB to conceptualize the various behaviors of ex-employees. These behaviors are discretionary, non-contractual reward requirements that contribute to organizational effectiveness. Second, using SIT as a framework, this study considers an LI perspective to illustrate how Ex-ECB is generated, which provides a proper perspective to explain how Ex-ECB occurs. Third, premised on SIT, the interaction between POP and PIS is posited as the antecedent of LI and Ex-ECB, which provides a comprehensive picture of the factors relating to LI and Ex-ECB. Fourth, the study identifies the CR between current and former organizations as a moderator, which affects the relationship between the LI and Ex-ECB. The study also explores how the interaction between POP and PIS affects Ex-ECB through LI, enriching the boundary conditions of how citizenship behavior is generated. Figure 1 illustrates the hypothesized research model.

## Study 1

### Ex-employee citizenship behavior and its constitutive definition

Organizational citizenship behavior has been widely studied because it helps improve organizational effectiveness (Khan et al., 2019; Pham et al., 2019; Zheng et al., 2021; Khan and Khan, 2022; Sa’adah and Rijanti, 2022). Given the boundary expansion of human resource management, various subjects, such as ex-employees, are increasingly being considered partial employees (Revilla-Camacho et al., 2015). In practice, a growing number of companies have taken measures for ex-employee management, such as organizing regular gatherings, providing ongoing perks, and distributing celebratory gifts (Masterson and Stamper, 2003). Ex-employees are increasingly becoming an indispensable part of organizational human resource management (Iyer et al., 1997; Sertoglu and Berkowitch, 2002; Somaya et al., 2008; Koc-Menard, 2009; Corredoira and Rosenkopf, 2010; Dokko and Rosenkopf, 2010; Farrow and Yuan, 2011; Herda and Lavelle, 2011; Carnahan and Somaya, 2013; Godart et al., 2014; Shipp et al., 2014; Tan and Rider, 2017). Given this context, OCB studies’ narrow focus on formal employees has been criticized by some researchers, arguing that such studies should include a wide range of other subjects (Groth, 2016). Specifically, many researchers advocate that OCB studies examine ex-employees, as they can provide various benefits to their former organizations (Somaya et al., 2008; Corredoira and Rosenkopf, 2010; Dokko and Rosenkopf, 2010; Carnahan and Somaya, 2013; Godart et al., 2014), and many ex-employees have accumulated in enterprises because of increased employee mobility (Dachner and Makarius, 2022). In response, the present study extends OCB to ex-employees, forming the concept of Ex-ECB.

This study defines Ex-ECB as discretionary behaviors which benefit the former organization but are not contractually rewarded by it. The first component of this constitutive definition, “discretionary,” suggests that the behavior is not an executable requirement of the role or behavior description, but a matter of personal choice (Organ, 1988). As no requirements are placed on ex-employees by their former organizations, their behaviors are personal and discretionary (Herda and Lavelle, 2011). The next part of the definition, “benefit the former organization,” implies that these behaviors will contribute to the former organization’s effectiveness. According to previous literature, ex-employees can provide their former organizations with business opportunities (Carnahan and Somaya, 2013) and knowledge (Corredoira and Rosenkopf, 2010). These, among other benefits, are important for improving organizational performance and effectiveness. The last segment, “not contractually rewarded,” suggests that these behaviors will not be recompensed by the former organization’s reward system directly or formally. This does not mean that ex-employees who develop citizenship behavior will be limited in any tangible return; importantly, such returns are not contractually guaranteed. Since employment termination includes employment contract invalidation, ex-employees’ behaviors are unrelated to contractual rewards.

### Measure development

The measure of Ex-ECB was developed following Hinkin’s (1998) tutorial. These steps included initial item generation, content validity assessment, item scaling, initial item reduction, internal consistency assessment, confirmatory factor analysis, and convergent validity. Subsequently, we constructed a nomological network of this concept, and nomological validity was assessed (section “Nomological network of Ex-ECB”).

First, initial items were generated. To ensure the success of initial item generation, we carefully considered the development of a well-articulated theoretical foundation that indicates the content domain for the new measure. Our goal was to develop measures that would sample the theoretical domain of interest to demonstrate content validity (Hinkin, 1998). Based on this principle, as well as previous research on OCB, we constructed a definition of Ex-ECB, as previously described. To identify the initial items for the construct of Ex-ECB, we conducted an interview study in April 2020. After ensuring voluntary participation and response confidentiality, 110 qualified MBA students at a large Chinese university were interviewed, all of whom had experience with voluntary termination. A lot of literature in the field of termination has focused on voluntary termination (Hom et al., 2017). Furthermore, forcibly terminated ex-employees were considered to be less qualified; therefore, this study focused only on voluntarily terminated ex-employees. Among the participants, 49 were men (44.5%) and 61 were women (55.5%). The average age of participants was 31.4 years, and their average tenure was 8.5 years. The participants had work experience in seven industry sectors across the Global Industry Classification Standard (GICS), and all of them had at least one voluntary termination experience. The initial items were developed using an inductive method (Hinkin, 1998). After we explained the concept of Ex-ECB, we asked participants to report their own or familiar acquaintances’ relevant behaviors. A total of 106,069 words from interview records were obtained. Three doctoral students from the Department of Human Resources Management were asked to develop initial items, according to Hinkin’s (1998) procedure. A total of 271 behavioral encodings were obtained, after the analysis of 75 interview records randomly selected by SPSS. The same behaviors were then merged, and irrelevant ones were deleted. Following the principle of more than 75% agreement, eight initial items were generated. By analyzing the results of the remaining 35 interview records, we observed that no new items were generated; thus, the eight items reached theoretical saturation.

Next, a content validity assessment was conducted. Content validity refers to how well a scale measures a given construct. We used a popular method, provided by Hinkin (1998), to assess content validity. We recruited 30 judges without previous knowledge of Ex-ECB and explained the concept to them. We then asked them to judge whether the above eight items conformed to the concept of Ex-ECB. Valid items in this step were chosen according to the principle of agreement of more than 75%. As a result of this assessment, two items were eliminated. Some judges were interviewed to determine why the two items were invalid. “Boomerang behavior” refers to an employee’s choice to return to a former employer. This concept was eliminated, as this behavior is often a single occurrence and does not necessarily benefit the former organization. Cooperative behavior was also eliminated, as the concept was too vague. Finally, six items were retained, as shown in Table 1.

**TABLE 1:** Items and factor loadings from confirmatory factor analysis.

Number	Items	Factor loadings
Ex-ECB1	Puts forward advice to benefit the former company (Voice behavior)	0.82
Ex-ECB2	Publicize the products or services of the former organization (Propaganda behavior)	0.90
Ex-ECB3	Transfer industry, market or experience information which conforms to law and ethics (Information transfer behavior)	0.80
Ex-ECB4	Conveys the positive side of the former organization to the outside to maintain a good organizational image (Maintaining corporate image behavior)	0.85
Ex-ECB5	Introducing new business or cooperate opportunities to the former organization (Business recommendation behavior)	0.77
Ex-ECB6	Introducing talents to the former organization (Talent recommendation behavior)	0.77

*N* = 81.

The third step was item scaling. Likert-type scales are the most frequently used in survey questionnaire research, and they have high compatibility for use in behavioral research (Hinkin, 1998). As such, the Likert 5-point scale was used to measure the above six items. Participants were asked to respond to these items on this scale (1 = strongly disagree, 2 = disagree, 3 = neither agree nor disagree, 4 = agree, 5 = strongly agree).

Next, the initial number of items was reduced. Once data were collected, factor analysis was conducted to further refine the new scales (Hinkin, 1998). The pretest samples were collected before the formal survey. As with the formal test samples, it was required that participants’ most recent termination was voluntary and that they held permanent employment at their former organizations. Participants were obtained using snowball sampling, through the alumni groups of three universities in China. Eligible indirect participants were obtained through direct participants. Two attention test questions and a final self-evaluation question of seriousness were used to screen out careless participants. One hundred data points were collected. In the pretest stage, 84 eligible participants were included, of which 46 were women (54.8%) and 38 were men (45.2%). One participant was aged 20 years or less (1.2%), 52 were aged 21–30 years (61.9%), 29 were aged 31–40 years (34.5%), and two were aged 41 years or above (2.3%). Of all the participants, 19 had not achieved a bachelor’s degree (22.6%), 59 had a bachelor’s degree (70.2%), and 6 had a master’s degree or above (7.2%). SPSS22.0 was used for analysis in the pre-test stage. The KMO was 0.90 (>0.70), and Bartlett’s test was significant (*p* < 0.001), indicating that the scale met factor analysis requirements. An exploratory factor analysis revealed that the six items were presented on one factor, with a contribution rate of variance reaching 72.6%. Each item’s factor loading was greater than 0.5, indicating that the internal structure was clear, the construction validity was high, and the contribution to the potential construction was ideal. All of these aspects conformed to the theoretical hypothesis of one-dimensional construction. Thus, all six items were retained.

Internal consistency was then assessed to determine the efficacy of our test measures. The higher the internal consistency, the more reliable the survey (Hinkin, 1998). Cronbach’s α was calculated to evaluate internal consistency and stability, and the results showed that it was 0.92, which is higher than the suggested minimum of 0.70. Thus, the internal consistency of the scale was verified.

Next, we assessed the factor structure’s goodness of fit by performing a confirmatory factor analysis (CFA) with 84 participants in the pretest stage (Hinkin, 1998). We used CFA and maximum likelihood estimation to test the proposed one-factor model which emerged from the exploratory factor analysis. The fit indices showed that a unidimensional model (single ex-employee citizenship factor) fit the data well. The comparative fit index (CFI) was 0.99, the standardized root mean square residual (SRMR) was 0.026, and the root mean square error of approximation (RMSEA) was 0.07, χ^2^/df = 1.38. These values were all at or above the recommended standards (e.g., Bagozzi et al., 1998). The items and standardized factor loadings for this CFA are shown in Table 1.

Finally, convergent validity was assessed to test how closely an item was related to other items measuring the same construct. To calculate convergent validity, we calculated average variance extracted (AVE) and composite reliability. The results revealed an AVE of 0.73 (>0.5) and composite reliability of 0.94 (>0.7). Convergent validity is achieved when the correlations between items of the measures are sufficiently high (Hinkin, 1998); thus, the high convergent validity of the scale was verified.

### Nomological network of ex-employee citizenship behavior

**FIGURE 2:**
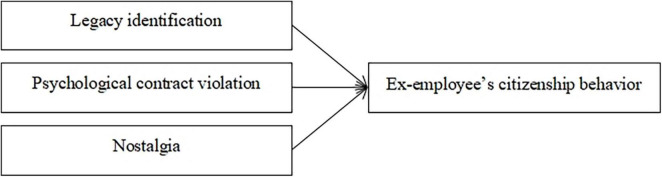
The nomological network among Ex-ECB, LI, PCV, and nostalgia.

Nomological validity was assessed according to the extent to which systematic support exists for theoretically proposed connections between Ex-ECB and related variables (Hinkin, 1998). According to SIT, SET, and previous nostalgia research, we summarized the nomological network of Ex-ECB (Figure 2).

First, we found that LI was positively related to Ex-ECB. According to SIT, people are likely to generate behaviors consistent with their identity (Ashforth and Mael, 1989). The more strongly a person identifies with an organization, the more he or she will be an active contributor to the organization (Van Dyne et al., 1994). Thus, ex-employees engage in more Ex-ECB when they identify with their former organization. Second, we found that psychological contract violation was negatively related to Ex-ECB. Based on the SET, when the ex-employee feels that the organization had “failed to fulfill the obligation” during employment tenure, a psychological contract violation (PCV) occurs and will persist after termination (Tian et al., 2022). This may trigger a strong negative emotional response, leading to a decrease in Ex-ECB. Third, we found that nostalgia was positively related to Ex-ECB. In this study, nostalgia refers to an ex-employee cherishing the “good old days” in their former organization (Bardon et al., 2015). According to previous research, nostalgia plays a significant role in explaining the positive effect of ex-employees on former organizations (Eury et al., 2018). Many theories posit that affection is a significant influencing factor of behavior (e.g., affective effects theory). Thus, we proposed that nostalgia is positively related to Ex-ECB.

To test nomological validity, snowball sampling was used to collect data. Direct participants were obtained from the alumni groups of three universities in China. Eligible indirect participants were obtained through direct participants. Each participant was informed of the study’s purpose and procedures, and they received a compensation of 5 RMB for each wave. We then distributed surveys at two time points, using WeChat (the most widely used social application in China). Three questions were set in the beginning, to identify the eligible participants. Subjects were required to have been full-time employees at their former organizations, and their most recent termination must have been voluntary. In the first wave, 210 participants were recruited. They participated in a survey on LI, PCV, and nostalgia. Fifteen days later, a second wave was conducted, and 181 participants were obtained. They participated in a survey on Ex-ECB and demographic information. A unique WeChat ID was used to match the two-wave surveys. Three attention test questions and a final self-evaluation question on seriousness were used to screen out careless participants. A total of 172 participants were included in this study. Among them, 90 were women (52.3%) and 82 were men (47.7%). Two were aged 20 or below (1.2%), 95 were aged 21–30 years (55.2%), 62 were aged 31–40 years (36.0%), and four were aged 41 years or above (2.3%). Forty-eight had not achieved a bachelor’s degree (27.9%), 98 had a bachelor’s degree (57.0%), and 26 had a master’s degree or higher (15.1%).

Participants were asked to score examples of Ex-ECB (six items, Cronbach’s α = 0.90, Time 2), LI (six items, Cronbach’s α = 0.89, Mael and Ashforth, 1992, Time 1), PCV (four items, Cronbach’s α = 0.91, Priesemuth and Taylor, 2016, Time 1), and nostalgia (three items, Cronbach’s α = 0.87, Hepper et al., 2012, Time 1). A 5-point Likert response format was used (from 1 = “strongly disagree” to 5 = “strongly agree”). Question items included “puts forward suggestions to benefit the former company” (Ex-ECB); “when someone praises my former organization, it feels like a personal compliment” (LI); “I feel betrayed by my former organization” (PCV), and “I feel nostalgic when I think about my former organization” (nostalgia).

**FIGURE 3:**
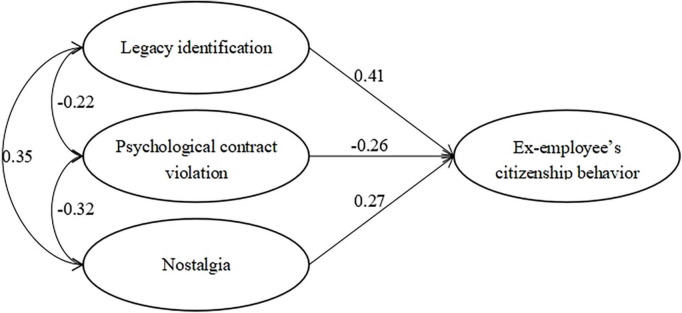
Results of path analysis for the nomological network.

Correlation analyses showed that Ex-ECB was positively and significantly correlated with LI (*r* = 0.59) and nostalgia (*r* = 0.51), and it was negatively and significantly correlated with PCV (*r* = –0.49) at moderate levels. In addition, we conducted path modeling using structural equation modeling (SEM) to test the hypothesized relationships. We specified the effects of LI, PCV, and nostalgia on Ex-ECB. The path model fit the data well (χ^2^ = 269.59, df = 44, *p* < 0.001, CFI = 0.94, RMSEA = 0.07). As shown in Figure 3, LI (γ = 0.41, *p* < 0.001), PCV (γ = –0.26, *p* < 0.001), and nostalgia (γ = 0.27, *p* < 0.01) were positively related to Ex-ECB.

## Study 2

### Theoretical basis and hypothesis development

#### Legacy identification and ex-employee citizenship behavior

Based on SIT, people are likely to generate behaviors consistent with their identity (Ashforth and Mael, 1989). The more strongly a person identifies with an organization, the more he or she will be an active contributor to the organization (Van Dyne et al., 1994). If ex-employees still identify with their former organizations, they will be inclined to consider their former organizations’ success or failure as their own. Thus, it is consistent LI for these ex-employees to exhibit Ex-ECB that benefits their former organization. Previous studies have supported this hypothesis. Iyer et al. (1997) indicated that LI may increase business recommendations. Mael and Ashforth (1992) suggested that former employees’ LI may increase their donations and recommendations. Eury et al. (2018) showed that if members’ LI is challenged, their contributing behaviors will also be challenged. Therefore, we proposed the following hypothesis:

H1: LI is positively related to Ex-ECB.

#### Perceived organizational prestige, perceived insider status, and legacy identification

According to SIT, the organization’s attributes closely relate to its members’ self-concept, because of social classification. Furthermore, people tend to enhance their self-concept (Hogg, 2006). Thus, the prestige of the organization will increase the identification of the organization by improving its member’s self-esteem (Ashforth and Mael, 1989; Hogg, 2006). For ex-employees, LI is also based on positive social classification. Ex-employees often maintain a positive self-concept (Hogg, 2006) when they believe that outsiders positively view their former organization; thus, they may “enjoy the glory reflected by their former organization” (Dutton et al., 1994) and act to improve their LI with it. POP refers to people’s consideration of how others view the organization to which they belonged (Shenkar and Yuchtman-Yaar, 1997). The higher the POP of the former organization, the greater the promotion of ex-employees’ self-esteem (March and Simon, 1958). Therefore, POP will have a positive impact on LI as a manifestation of “how others view the organization.” Research on the relationship between prestige and identification was first studied by March and Simon in 1958. Many subsequent studies have provided direct support for the relationship between POP and LI (Mael and Ashforth, 1992; Smidt et al., 2001; Eury et al., 2018). For example, when an organization’s prestige is damaged by a scandal, the LI of its former members will be challenged (Eury et al., 2018).

Furthermore, according to SIT, social classification cannot affect identification independently (Hogg, 2006). Interaction, communication, and interdependence between individuals and their organizations are the psychological basis for LI (Hogg, 2006). Traditionally, leaving an organization meant that employees are disconnected from the organization, and employees are transformed from insiders to outsiders (Scott, 2003). Both parties stop interacting, communicating, and being interdependent. Thus, ex-employees do not have a psychological basis for LI. However, an increasing number of organizations are actively connecting with former employees (Masterson and Stamper, 2003). These supporting measures are a prerequisite for individuals to perceive insider status (Stamper and Masterson, 2002). With PIS, an individual perceives a sense of belonging to and acceptance by an organization. PIS reflects an individual’s sense of membership as an “insider” of the organization (Stamper and Masterson, 2002). Stamper and Masterson (2002) emphasized that the perception of being an insider is distinct from actual inclusion (Stamper and Masterson, 2002), which makes it possible for ex-employees to perceive insider status despite not actually being included by their former organization. A high PIS leads to positive interactions, communication, and dependency between ex-employees and their former organizations. These aspects provide a profound psychological basis for LI and thus affect it interactively with PIS. Considering these points, the following hypothesis was proposed.

H2: The interaction between POP and PIS is positively related to LI, such that LI identification is highest when both perceived POP and PIS are high.

#### The mediating role of legacy identification

According to SIT, the distinctiveness of a group is closely related to its members’ self-concept, and people are inclined to maintain a positive self-concept (Ashforth and Mael, 1989). Thus, LI is positively impacted by POP, which manifests in “how others view the organization.” However, the identification does not completely result from organizational prestige. The relationship quality between ex-employees and former organizations is the psychological basis of LI (Hogg, 2006). Thus, the interaction between POP and PIS is positively related to LI. Moreover, because ex-employees tend to perform behaviors consistent with their identification (Ashforth and Mael, 1989), LI impacts their citizenship behavior. Based on H1 and H2, the following hypothesis was proposed.

H3: POP and PIS are indirectly and interactively related with Ex-ECB, through LI. Specifically, the positive and indirect relationship between POP and Ex-ECB is more pronounced when PIS is high.

#### The moderating role of the cooperative relationship

Social identity theory holds that by awakening or inhibiting a certain identity, identity salience will impact the relationship between a certain identification and subsequent behaviors (Marin et al., 2009; Wood and Caldas, 2009). Specifically, individuals may identify with multiple groups, and the salience of these identities may vary to different degrees (Serpe, 1994). When the salience of a specific identity is high, individuals tend to generate perceptions, thoughts, emotions, and behaviors according to the typical norms, role models, and patterned views of group members (Randel, 2002). With high identity salience, the relationship between social identification and subsequent behaviors is stronger. Therefore, identity salience plays a moderating role in the relationship between social identification and subsequent behaviors. However, the degree of identity salience does not indicate the degree of social identification (Wood and Caldas, 2009). In a post-employment context, the CR between current and former organizations is an important factor, which affects the relationship between ex-employees and their former organizations (Somaya et al., 2008; Campbell et al., 2012; Carnahan and Somaya, 2013; Bermiss and Greenbaum, 2016; Mawdsley and Somaya, 2016). Specifically, if a CR exists, LI will have high legitimacy in the context of the current organization, because LI can produce resources, such as business cooperation. Thus, LI tends to be acquired or supported by the current organization (Somaya et al., 2008; Corredoira and Rosenkopf, 2010; Carnahan and Somaya, 2013). In this case, the LI salience is high, and the relationship between LI and Ex-ECB is stronger. Otherwise, the LI of the former organization is often regarded as a threat for the current organization to discourage or even avoid (Somaya et al., 2008; Bardon et al., 2015). In this case, the LI salience is low; even if ex-employees have a high LI, it may still be suppressed. The relationship between LI and Ex-ECB will then be weaker. Therefore, we proposed the following hypothesis:

H4: The positive relationship between LI and Ex-ECB is moderated by the CR between the current and former organizations. Furthermore, the positive relationship is stronger when a CR is present.

Following H1, H2, H3, and H4, the following hypothesis was proposed:

H5: The indirect positive effect of the interaction between POP and PIS on Ex-ECB through LI is moderated by the CR between the current and former organizations, such that the mediating effect is more pronounced when a CR is present.

### Materials and methods

#### Samples and procedures

This study used snowball sampling to collect data. Direct participants were obtained through the alumni groups of three universities in China. Eligible indirect participants were referred through direct participants. Each participant was informed of the purpose and procedure of the study, and they received five RMB compensation for each wave. We then distributed surveys at two time points in December 2021 using WeChat. Three questions were positioned at the beginning of the survey to identify the eligible participants. To be eligible for the study, the subjects’ most recent termination must have been voluntary and from a full-time employment position. In the first wave of the study, 279 participants were recruited. They participated in a survey assessing demographic information, POP, PIS, and LI. Fifteen days later, the second wave was conducted. A total of 236 participants were included in this study. They participated in a survey assessing Ex-ECB and CR. A unique WeChat ID was used to match the two-wave surveys. Three attention test questions and a final self-evaluation question on seriousness were used to screen out careless participants. A total of 218 participants were retained. As the eligible data sample was too small, the same procedures were used to collect additional data in August 2022. A total of 120 participants were recruited during the first wave, and 101 were retained after the second wave. The unique WeChat ID was used to match the two-wave surveys and to eliminate subjects who had already participated in our December research. A total of 73 eligible participants were included. In total, 291 data points were collected. Among them, 139 were women (47.8%) and 152 were men (52.2%). Four were aged 20 years or below (1.4%), 165 were aged 21–30 years (56.7%), 100 were aged 31–40 years (34.4%), and 20 were aged 41 years or above (6.9%). Of these, 93 had not achieved a bachelor’s degree (32.0%), 181 had a bachelor’s degree (62.2%), and 17 had a master’s degree or above (5.8%).

#### Measures

Perceived organizational prestige, PIS, and LI were measured using maturity scales written in English. Four doctoral students majoring in Human Resource Management conducted a “back translation” procedure to generate the Chinese version. Study 1 developed the Ex-ECB scale. All scales were revised based on a pre-test (84 participants). All scales adopted a 5-point Likert scale, with “1” meaning “very inconsistent” and “5” meaning “very consistent.” Finally, we used CR as a two-category moderating variable.

##### Perceived organizational prestige

To measure POP, we referred to the scale of Mael and Ashforth (1992). However, we deleted items “I would be proud to have my children attend my former organization” and “Seeking to advance my career should downplay my association with my former organization,” because the factor loadings for these were less than 0.5. The former item was deleted because it was not relevant to the state of Chinese enterprise. Many prestigious companies in China, such as technology firms, are relatively new. Simultaneously, many of the prestigious companies where parents once worked have disappeared because of China’s reform and development. Thus, there is little opportunity for Chinese parents and children to work at the same company. The latter item was deleted because it did not have a straightforward relationship with POP in the Chinese context. Highlighting the association with a former organization may be harmful to an employee’s present career (especially when there is no CR between the current and former organizations), regardless of organizational prestige. Thus, reaction to the former organization is not a plausible item of POP. Six items remained, including “People in my community think highly of my former organization.” The Cronbach’s alpha was 0.90.

##### Perceived insider status

To measure PIS, the Stamper and Masterson (2002) scale was rewritten for the context of post-employment. We deleted the item “I feel like I am an ‘outsider’ at this organization after I have left,” since the factor loading was less than 0.5. Through the interviews, we realized that this item was not suitable for the post-employment context. For most individuals, termination means they have become legal outsiders; thus, this item induced participants to choose a higher score despite having high PIS. Five items remained, including “Although I have left, I still feel that I was a part of my former organization.” The Cronbach’s alpha was 0.93.

##### Legacy identification

To measure LI, Mael and Ashforth’s (1992) one-dimensional scale was rewritten for a post-employment context. This scale has been used to measure alumni’s identification with their alma mater, as well as ex-employees’ identification with accounting firms, and it has high reliability and validity (Mael and Ashforth, 1992; Iyer et al., 1997). The scale included six items, including “When someone praises my former organization, it feels like a personal compliment.” The Cronbach’s alpha was 0.93.

##### Cooperative relationship

The CR between the current and former organizations was a categorical variable. This was measured by one question: “Does your current organization have a CR with your former organization?” The answer options were “Yes” or “No.”

##### Control variables

The control variables were proposed based on previous studies. First, the former organizational tenure and time-lapse might affect the LI (Wood and Caldas, 2009). Therefore, these two variables were measured with the questions “How long did you work for your former company?” and “How long is it since you left your former company?” In addition, the present tenure was measured by the question “How long have you worked in the current company?” Finally, this study was controlled for individual characteristics, such as gender, age, and education (Mael and Ashforth, 1992; Hogg, 2006).

### Results

Discrimination validity and common method biases were first calculated. Descriptive statistics and correlation analyses were then performed. Finally, hierarchical regression, simple slope analysis, and the bootstrap method were applied to test our hypotheses.

#### Confirmatory factor analysis and common method biases test

To test the discrimination validity of each variable, AMOS17.0 was used for CFA. Table 2 shows that the four-factor model yielded a better fit than the alternative models.

**TABLE 2:** Confirmatory factor analysis for discriminant validity.

Variable	CMIN	df	CMIN/df	RMSEA	CFI	GFI	TLI	IFI
Four-factor model	530.13	224	2.37	0.07	0.94	0.86	0.94	0.93
Three-factor model	1499.71	227	6.61	0.14	0.76	0.59	0.73	0.76
Two-factor model	2106.89	229	9.20	0.17	0.64	0.52	0.60	0.64
Single factor model	3376.53	230	14.68	0.22	0.40	0.38	0.34	0.40

*N* = 291, the four-factor model includes POP, PIS, LI, and Ex-ECB; the three-factor model includes POP + PIS, LI, and Ex-ECB; the two-factor model includes POP + PIS + LI and Ex-ECB; the single factor model includes POP + PIS + LI + Ex-ECB.

To reduce common method bias, process and statistical controls were performed. Process control was ensured by the anonymity of our data collection methods, as well as the inclusion of reverse questions and screening questions. To ensure statistical control, Harman’s single-factor test was performed in the data analysis stage. The first factor explained 34.86% of the variance, which was less than 40% (Podsakoff and Organ, 1986). Thus, no significant common method bias was present in this study.

#### Descriptive statistics and correlation analysis

The descriptive statistical analysis results are shown in Table 3. POP, PIS, LI, and Ex-ECB were all positively correlated at a significance level of 0.001. Among them, POP was positively correlated with LI (*r* = 0.35, *P* < 0.001), POP was positively correlated with Ex-ECB (*r* = 0.28, *P* < 0.001), PIS was significantly positively correlated with LI (*r* = 0.48, *P* < 0.001), PIS was significantly positively correlated with Ex-ECB (*r* = 0.22, *P* < 0.001), LI was significantly positively correlated with Ex-ECB (*r* = 0.35, *P* < 0.001), and CR was significantly positively correlated with Ex-ECB (*r* = 0.15, *P* < 0.05). In addition, the interaction between POP and PIS was significantly positively correlated with LI (*r* = 0.11, *P* < 0.05), and the interaction between LI and CRs was significantly positively correlated with Ex-ECB (0.17, *P* < 0.01).

**TABLE 3:** Means, standard deviations, and intercorrelations.

	Mean	*SD*	1	2	3	4	5	6	7	8	9	10	11	12
1. Gender	1.52	0.50	1											
2. Age	30.74	5.91	−0.19**	1										
3. Education	3.66	0.78	0.07	−0.18**	1									
4. Former tenure	2.71	1.80	−0.17**	0.60***	–0.11	1								
5. Time-lapse	2.04	1.96	–0.03	0.46***	−0.20**	0.34***	1							
6. Present tenure	1.84	1.92	–0.00	0.46***	−0.15*	0.32***	0.92***	1						
7. POP	3.48	0.85	–0.05	0.10	0.16**	0.05	–0.02	0.02	1					
8. PIS	3.38	1.09	0.15*	–0.09	0.10	0.02	0.04	0.04	0.15**	1				
9. PIS*POP	0.14	1.02	0.10	–0.05	0.02	–0.03	0.02	0.03	−0.11*	0.06	1			
10. LI	3.20	1.03	0.06	–0.07	0.21***	–0.05	–0.07	–0.06	0.35***	0.48***	0.11*	1		
11. CR	0.23	0.42	0.02	0.12*	−0.14*	0.12*	0.06	0.08	0.08	0.26***	−0.18**	0.18**	1	
12. LI*CR	0.08	0.42	–0.01	–0.08	–0.03	–0.02	–0.03	0.02	–0.08	0.02	0.23***	–0.03	0.24***	1
13. Ex-ECB	3.42	1.03	0.03	0.10	–0.06	0.03	–0.00	–0.02	0.28***	0.22***	0.18**	0.35***	0.15*	0.17**

*N* = 291, *p < 0.05, **p < 0.01, ***p < 0.001 (two-tailed). Gender was coded as “1” for men and “2” for women. Education was coded as “1” for “high school diploma or below,” “2” for “college diploma,” “3” for “bachelor’s degree,” and “4” for “master’s degree or above”.

#### Hypothesis test

A hierarchical regression analysis was conducted using SPSS 22.0 to verify H1. As demonstrated by Model 3 in Table 4, LI was positively related to Ex-ECB (β = 0.38, *p* < 0.001). Thus, H1 was verified.

**TABLE 4:** Results of multiple regression analysis.

	LI		Ex-ECB		
		
	Model 1	Model 2	Model 3	Model 4	Model 5
Gender	–0.00	–0.01	0.04	0.04	0.05
Age	0.03	0.03	0.16*	0.16*	0.19*
Education	0.12*	0.11*	−0.12*	–0.11	−0.11*
Former tenure	–0.06	–0.06	–0.03	–0.04	–0.04
Time-lapse	–0.00	–0.00	0.09	0.10	0.11
Current tenure	–0.05	–0.06	–0.17	–0.18	–0.19
POP	0.43***	0.43***			
PIS	0.26***	0.28***			
POP*PIS		0.12*			
LI			0.38***	0.36***	0.38***
CR				0.06	0.00
LI * CR					0.19***
R-square	0.33	0.34	0.157	0.160	0.20
F value	17.13***	16.13***	7.56***	6.73***	7.55***

*N* = 291, *p < 0.05, ***p < 0.001 (two-tailed).

**FIGURE 4:**
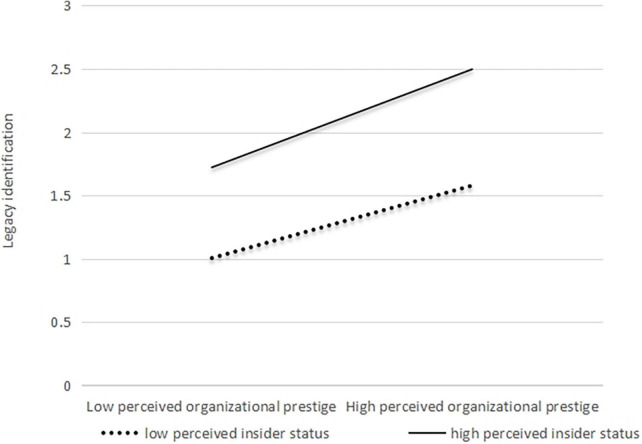
Interactive relationship of POP and PIS with LI.

Hierarchical regression and simple slope analyses were conducted to verify H2. The interaction term was calculated after the two variables were mean-centered to eliminate collinearity. Model 2 in Table 4 shows the interaction between POP and PIS, which was positively related to LI (β = 0.12, *p* < 0.05). To better interpret the moderating effect, following Cohen and Cohen (1983), we defined high- and low-PIS as plus and minus one standard deviation from the mean. As shown in Figure 4, LI is highest when both POP and PIS are high. Thus, H2 was verified.

H3 proposes a conditional indirect relationship (Preacher et al., 2007), whereby the strength of the indirect link between POP and Ex-ECB *via* LI is dependent on PIS. The pattern of the results for H1 and H2 offers tentative support for this complex association. To corroborate this conclusion, we used the bootstrap method to verify the mediation effect. As shown in Table 5, the index of moderated mediation (Hayes, 2015) was statistically significant, as indicated by a 95% CI (based on 5,000 resamples) that did not include zero (estimate = 0.04, *SE* = 0.02, 95%CI = 0.003, 0.08). These results indicate that PIS moderates the indirect association between POP and Ex-ECB *via* LI. As shown in Table 6, the conditional indirect relationship was most pronounced for ex-employees with higher PIS (+1 SD: *B* = 0.15, *SE* = 0.05, 95%CI = 0.07, 0.25). These findings supported H3.

**TABLE 5:** Index of conditional indirect model.

Index	BootSE	BootLLCI	BootULCI
0.04	0.02	0.003	0.08

*N* = 291, bootstrap sample *n* = 5,000; LLCI Lower/upper limit of 95% CI.

**TABLE 6:** Results of conditional indirect effect.

PIS	Effect	BootSE	BootLLCI	BootULCI
–1 SD	0.064	0.024	0.020	0.116
Mean	0.105	0.029	0.056	0.169
+1 SD	0.145	0.046	0.068	0.247

*N* = 291, bootstrap sample *n* = 5,000; LLCI Lower/upper limit of 95% CI.

**FIGURE 5:**
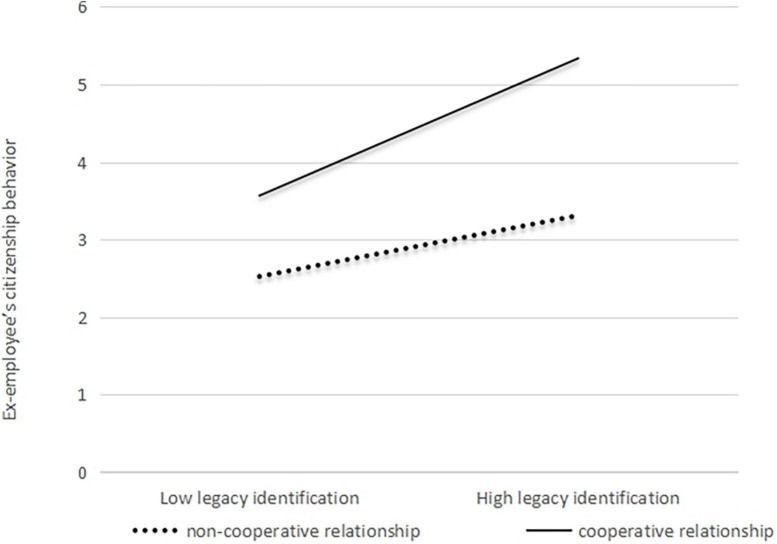
Moderating effect of CR on the relationship between LI and Ex-ECB.

A hierarchical regression analysis was used to verify H4. The results of the analysis are presented in Table 4. As shown in Model 5, the interactive term has a significant positive impact on Ex-ECB (β = 0.19, *p* < 0.001). Furthermore, the simple slope analyses shown in Figure 5 suggest that the relationship between LI and Ex-ECB is stronger when a CR exists between the current and former organizations. Thus, H4 was verified.

H5 proposed that the positive effect of the interaction between POP and PIS on Ex-ECB through LI is moderated by the CR between the current and former organizations, such that the mediating effect is stronger when a CR is present. The pattern of results from H1, H2, H3, and H4 offers tentative support for this complex association. To corroborate this conclusion, the bootstrap method was used to verify this effect. As shown in Table 7, the respective index of moderated mediation (Hayes, 2015) was statistically significant, as indicated by a 95% CI (based on 5000 resamples) that did not include zero (estimate = 0.06, *SE* = 0.03, 95%CI = 0.01, 0.13). These results indicate that the CR between the current and former organizations moderated the indirect association between the interaction term (POP and PIS) and Ex-ECB *via* LI. As shown in Table 8, the conditional moderated mediation relationship was more pronounced for ex-employees with a CR at every level of PIS (–1 SD: *B* = 0.15, *SE* = 0.06, 95%CI = 0.05, 0.28; Mean: *B* = 0.24, *SE* = 0.06, 95%CI = 0.14, 0.37; +1 SD: *B* = 0.33, *SE* = 0.09, 95%CI = 0.18, 0.53) than for ex-employees with non-CRs (–1 SD: *B* = 0.04, *SE* = 0.02, 95%CI = 0.01, 0.08; Mean: *B* = 0.07, *SE* = 0.03, 95%CI = 0.02, 0.13; +1 SD: *B* = 0.09, *SE* = 0.04, 95%CI = 0.03, 0.19). These findings supported H5.

**TABLE 7:** Index of the conditional moderated mediation model.

Index	BootSE	BootLLCI	BootULCI
0.060	0.031	0.006	0.128

*N* = 291, bootstrap sample *n* = 5,000; LLCI Lower/upper limit of 95% CI.

**TABLE 8:** Result of the conditional moderated mediation effect.

CR	PIS	Effect	BootSE	BootLLCI	BootULCI
0	–1 SD	0.040	0.019	0.008	0.084
	Mean	0.066	0.028	0.020	0.129
	+1 SD	0.091	0.042	0.025	0.189
1	–1 SD	0.146	0.059	0.047	0.278
	Mean	0.237	0.060	0.140	0.371
	+1 SD	0.329	0.088	0.179	0.528

*N* = 291, bootstrap sample *n* = 5,000; LLCI Lower/upper limit of 95% CI.

## Discussion

### Research conclusion

The termination of employment is not the end of the organization-employee relationship. Ex-employees can provide various benefits to their former organizations (Somaya et al., 2008; Corredoira and Rosenkopf, 2010; Dokko and Rosenkopf, 2010; Carnahan and Somaya, 2013; Godart et al., 2014). Moreover, as employee mobility increases, companies accumulate several former employees (Dachner and Makarius, 2022). Therefore, research on ex-employees’ contribution behavior, as well as how it is generated, is important in enabling firms to make use of their ex-employee resources effectively and improve organizational efficiency. Based on previous research on OCB, this study extends its focus to include ex-employees and develops the concept of Ex-ECB, to capture various former-organizational contribution behaviors. In addition, we explored how Ex-ECB is generated, based on SIT. Our findings in Study 1 suggest that Ex-ECB is reasonably defined as discretionary behaviors which benefit the formal organization but are not contractually rewarded by it. This measure contained six items, including “Offers advice to benefit the former company.” Our findings in Study 2 suggest that LI was positively related to Ex-ECB; the interaction between POP and PIS was positively related to LI; POP and PIS were indirectly and interactively related to Ex-ECB through LI; the positive relationship between LI and Ex-ECB was moderated by the CR between the current and former organizations; and the indirect positive effect of the interaction between POP and PIS on Ex-ECB through LI is moderated by the CR between the current and former organizations.

### Theoretical implications

First, most research on ex-employees’ contributions exists in the field of organizational strategy (Somaya et al., 2008; Corredoira and Rosenkopf, 2010; Dokko and Rosenkopf, 2010; Carnahan and Somaya, 2013; Godart et al., 2014). However, these studies place no conceptual value on personal choice, although it is ex-employees’ personal choice that realizes these values. The present study further explored ex-employees’ contributions in a micro-field of personal behaviors. Specifically, it extended the concept of OCB to include ex-employees and posited the concept of Ex-ECB to conceptualize ex-employees’ various behaviors. In addition, we developed the measurement of Ex-ECB according to a strict tutorial (Hinkin, 1998). In this measurement, six items were identified: voice, propaganda, information transfer, corporate image maintenance, business recommendation, and talent recommendation. The findings of this study compensate for the lack of research on ex-employees’ behaviors which could directly provide benefits to their former organizations. Simultaneously, the extension and innovation of OCB in the novel context of post-employment further develops the academic understanding of OCB.

Furthermore, the interaction between POP and PIS was posited as an antecedent of LI and Ex-ECB. These findings concur with SIT, showing that the identification resulted from neither organizational prestige nor social interaction and interdependence, such as PIS (Hogg, 2006). Instead, identification was a consequence of the interaction of POP and PIS. This understanding provides a comprehensive picture of the factors related to LI and Ex-ECB.

We also found that the CR between current and former organizations strengthened the relationship between LI and Ex-ECB, as well as the indirect positive effect of the interaction between POP and PIS on Ex-ECB through LI. These findings concur with previous research showing that identification is not related to an individual’s behaviors, but rather the relationship between identification and behaviors depends on identity salience (Hogg, 2006). CRs, as a specific form of identity salience in the context of post-employment (Tian et al., 2022), moderate the relationship between LI and Ex-ECB. Therefore, these results further our understanding of the boundary conditions under which LI may foster Ex-ECB.

### Practical implications

First, this study calls on organizations to monitor Ex-ECB. Although some excellent enterprises’ management practices lead to theories, many companies remain unaware of beneficial ex-employee behaviors; some do not even include ex-employees in their human resource management. This study explores the phenomenon of Ex-ECB and identifies six forms (voice, propaganda, information transfer, corporate image maintenance, business recommendation, and talent recommendation). Thus, this study may educate companies about the benefits of Ex-ECB.

This study also found that POP and PIS influence Ex-ECB through LI. Therefore, to improve LI and finally improve Ex-ECB, enterprises should pay attention to improving the POP and PIS of ex-employees. To improve POP, enterprises may institute efforts such as hiring high-profile leaders (Love et al., 2017), being cautious when hiring employees who may up the risk of corporate scandals (Eury et al., 2018), and adopting a gentle approach when firing employees (Love and Kraatz, 2009). To improve PIS, the enterprises could make efforts to establish post-employment communities, regularly hold meetings, provide employment consultation or help services, distribute birthday or holiday blessings and souvenirs, or maintain job numbers. In addition, since the exit process plays a significant role in reshaping the relationship between organizations and their ex-employees (Mai et al., 2016), the measures taken by the organization during this time are of great importance. Organizations should take a friendly approach to enhance the psychological base from which PIS originates.

Finally, to improve the efficiency of ex-employee management, enterprises should establish a robust external cooperation network and pay attention to the relationship between ex-employees’ current and former organizations. When a CR exists, an ex-employee has a more salient LI with their former organization, and they are more inclined to return resources. If a CR is not present, employers may suppress an employee’s LI with former organizations.

### Limitations and future research directions

First, this study explains how Ex-ECB is generated from LI. This is an important perspective in explaining Ex-ECBs and is lacking in the social network of former organizations, which has irreplaceable significance for how Ex-ECB is generated. In addition, further studies may want to explain Ex-ECB in the context of other theoretical perspectives, such as mourning (Walsh and Bartunek, 2012) and social exchange (Herda and Lavelle, 2011). Future studies may also explore other potential mechanisms to obtain a comprehensive understanding of how Ex-ECB is generated.

Moreover, like OCB, ex-employees who carry out citizenship behaviors are also faced with the distinction of being a “good soldier” or “good actor” (Donia et al., 2016). Although ex-employees’ good behavior can also bring positive benefits to the former organization, it contributes far less than “good soldier” behavior, because of its self-interested nature. Therefore, future research may distinguish between these two types of citizenship behavior, to obtain a more accurate and in-depth understanding of this concept.

Furthermore, this study identifies CRs as the boundary condition of the relationship between LI and Ex-ECB. It also considers the existence of a CR between the current and former organizations as an important factor. However, the relationships between former and current organizations can vary and become complicated. For example, they may cooperate in some fields but be competitive in other domains. Our measurement captured whether any CR existed between the current and former organizations. However, this definition failed to capture more complicated features of this concept, such as the degree and aspect of the cooperation. We will pursue this research direction, and we anticipate exploring the complexities of the CR between current and former organizations in the following studies.

Additionally, it is worth studying how Ex-ECB influences current organizations. Role conflict theory holds that individual participation in one role activity will inevitably lead to the reduction of time and energy to participate in another role activity; therefore, Ex-ECB would consume time and energy that would otherwise be contributed to the current organization (Rizzo et al., 1970). In contrast, role accumulation theory holds that individuals can participate in multiple role activities simultaneously, which can be mutually beneficial (Sieber, 1974). This study provides a perspective on concerns about CR. However, because of their complex nature, complementary employee roles warrant further investigation.

We also conducted scale development strictly according to Hinkin’s (1998) scale development procedure, and various tests showed high scale reliability and validity. However, other OCB measures with multiple dimensions (e.g., Farh et al., 1997; Groth, 2016) may better demonstrate specific behaviors. Moving forward, the scale could be further developed into a multi-dimensional measure.

Finally, the data used in this study were obtained from a single source. Although this study adopted a two-wave approach, it is still at risk of common method bias. In future research, multisource evaluation methods may be adopted; this approach would also reduce the problem of social approval in self-reported data.

## Data availability statement

The raw data supporting the conclusions of this article will be made available by the authors, without undue reservation.

## Ethics statement

Ethical review and approval were not required for the study on human participants, in accordance with the local legislation and institutional requirements. The subjects provided their written informed consent to participate in this study. Written informed consent was obtained from the individuals for the publication of any potentially identifiable images or data included in this article.

## Author contributions

ZT, QY, SQ, and YG: research design and revising the manuscript. ZT: data collection and writing of the original draft. ZT and SQ: data analysis. All authors contributed to the article and approved the submitted version.
